# Supplementary material for: Recreation of an antigen-driven germinal center in vitro by providing B cells with phagocytic antigen
Source: Commun Biol. 2023 Apr 20;6:437. doi: 10.1038/s42003-023-04807-0 (PMC10119099; doi:10.1038/s42003-023-04807-0)
Supplement: Supplementary file 2 — Supplementary Information-New [file 42003_2023_4807_MOESM2_ESM.pdf]

SUPPLEMENTARY INFORMATION

Recreation of an antigen-driven germinal center in vitro by providing B cells with phagocytic antigen

Ana Martínez-Riaño[1*], Pilar Delgado[1*], Rut Tercero[1*], Sara Barrero[1], Pilar Mendoza[1], Clara L. Oeste[1], David Abia[1], Elena Rodríguez-Bovolenta[1], Martin Turner[2] and Balbino Alarcón[1#]

[1]Centro de Biologia Molecular Severo Ochoa, CSIC-UAM, 28049 Madrid, Spain

[2]Immunology programme, The Babraham Institute, Babraham Research Campus, Cambridge CB22 3AT, United Kingdom

[#]To whom correspondence should be addressed at balarcon@cbm.csic.es

*Equally contributing authors

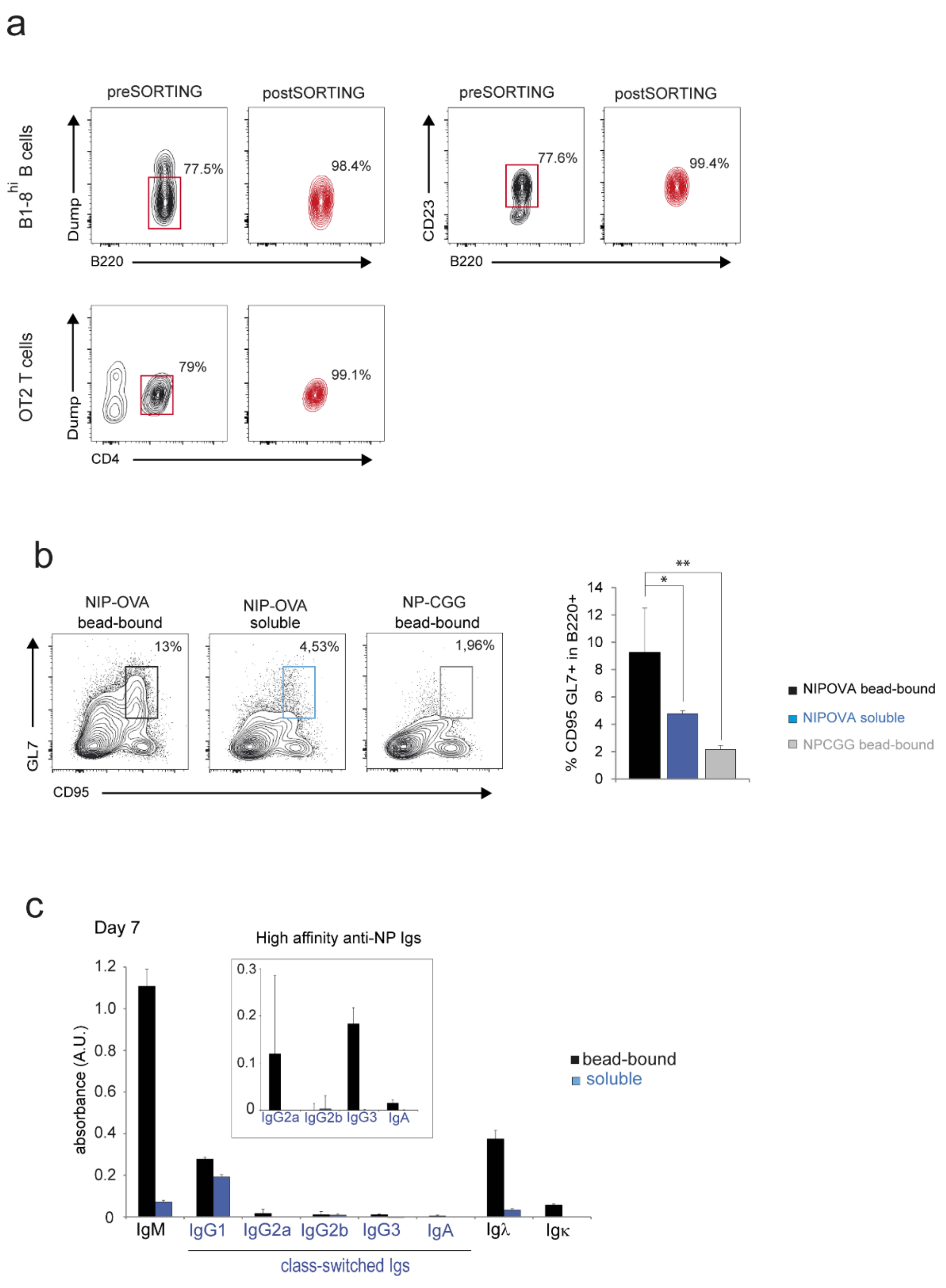

Figure S1. Generation of a GC reaction by phagocytic B cells and helper T cells does not require a third cell type. **a,** Sorting of naïve follicular B cells from B1-8^{hi} WT mice and of naïve CD4^{+} T cells from OT-2 transgenic mice. The Dump channel contain the CD11b^{+} and CD43^{+} cells (for follicular B cell sorting) and the B220^{+}, CD11b^{+}, CD8^{+}, NK1.1^{+}, F480^{+} cells (for CD4^{+} T cells). **b,** Sorted B and T cells as in a were incubated with either beads coated with NIP-OVA or NP-CGG (3:1 bead/B cell ratio), or with 100 ng/ml soluble NIP-OVA for 4 days at a 1:1 B/T cell ratio. Flow cytometry contour-plots to the left show the appearance of a double positive (CD95^{+} GL7^{+}) population in gated B220^{+} B cells. Bar plot to the right represents mean ± S.D. (n = 3): * p<0.05; ** p<0.005 (unpaired Student's t test). **c,** Detection of high-affinity anti-NP Igs in supernatants from B and T cell cultures prepared as in a and b incubated for 7 days (beads coated with NIP-OVA or soluble NIP-OVA). Data represent the mean ± S.D. (n = 3).

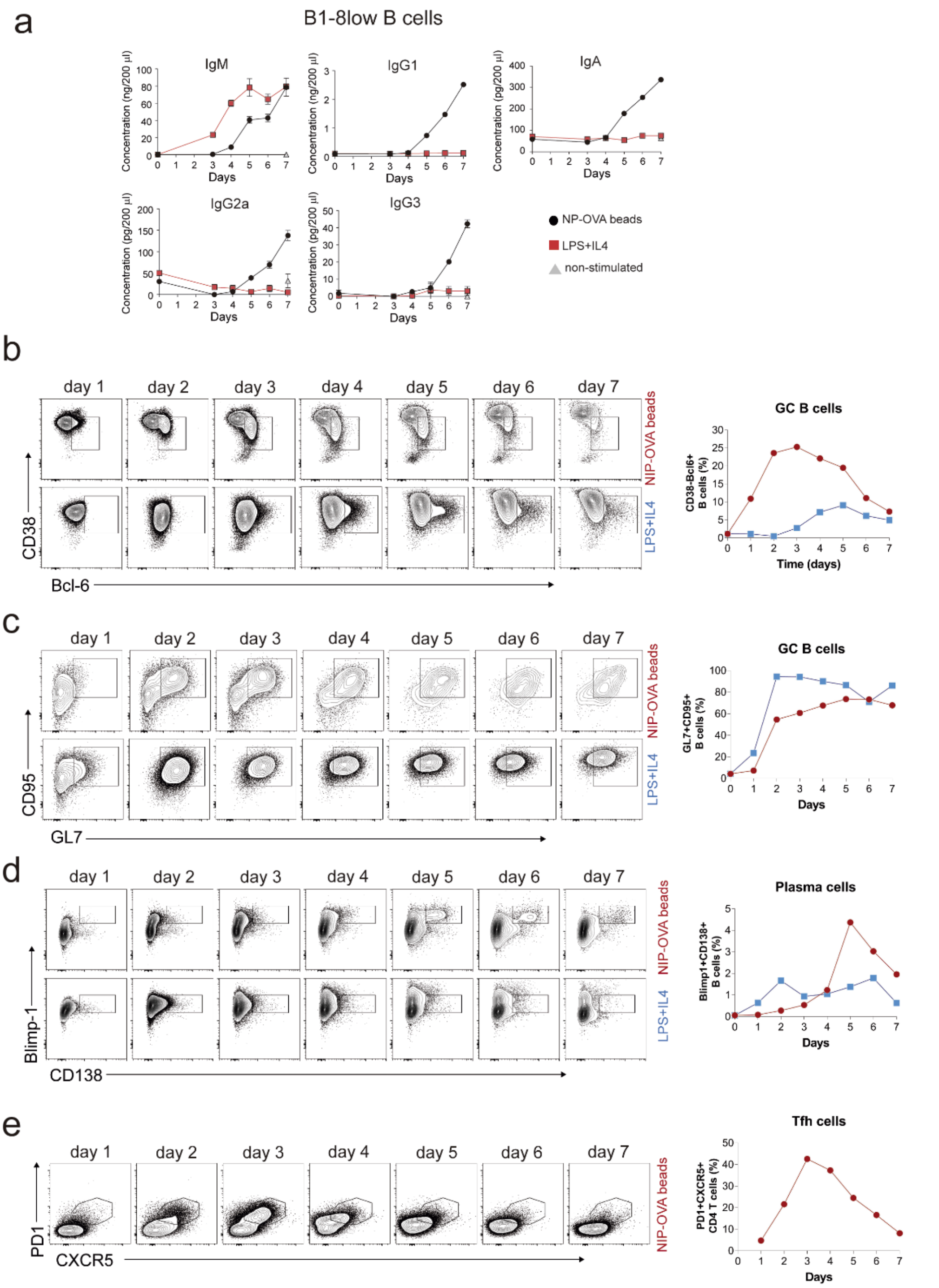

Figure S2. B cells undergo Ig class switching *in vitro* when stimulated with bead-bound antigen regardless the original affinity of the BCR expressed by naïve B cells. **a,** Detection of high-affinity anti-NP Igs in supernatants of B1-8^{low} B cells stimulated with bead-bound (black) NIP-OVA together with OT-2 T cells for the indicated number of days. In parallel, B1-8^{low} B cells were stimulated with LPS plus IL-4 as a polyclonal stimulus, in the absence of OT-2 T cells (red). Data represent the mean ± S.D. (n = 3). **b,** The percentages of GC B cells were calculated according to CD38 downregulation and expression of intracellular Bcl-6 by gated $B220^+$ B cells. **c,** The percentages of GC B cells were additionally calculated according to the expression of CD95 and GL7 by gated $B220^+$ B cells. **d,** The percentages of plasma cells were calculated according to the expression of CD138 and intracellular Blimp-1 by gated $B220^+$ B cells. **e,** Differentiation of OT-2 CD4+ T cells into Tfh was followed according to the expression of CXCR5 and PD1 markers. Line plots to the right represent the values of a single culture per timepoint.

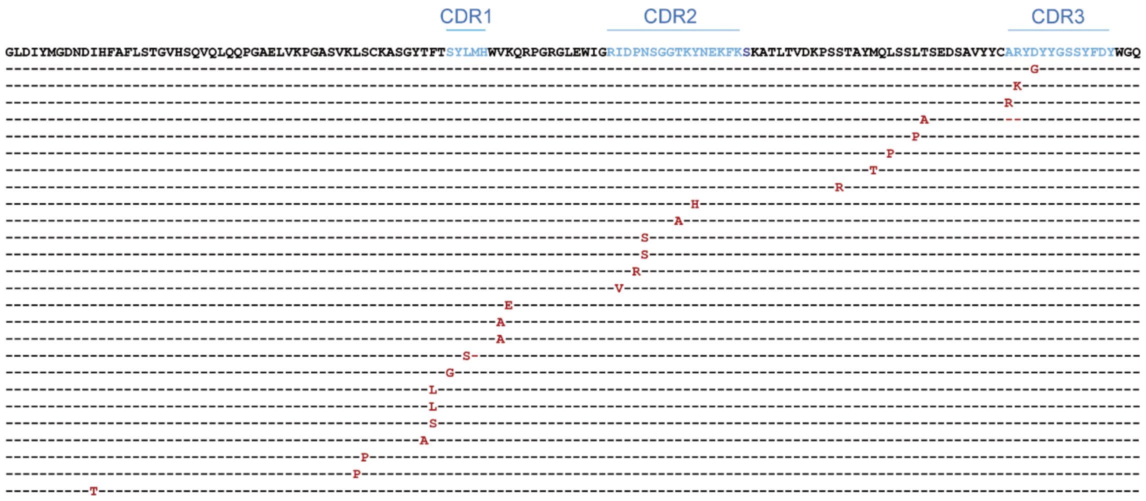

Figure S3. Generation of somatic mutations in IgH V genes in conditions of GC formation *in vitro*. Sequences of the B1-8V_H genes with amino acid replacement mutations detected by SANGER sequencing in B1-8[hi] B cells from Fig. 5b, depicting their distribution along the three CDR regions.

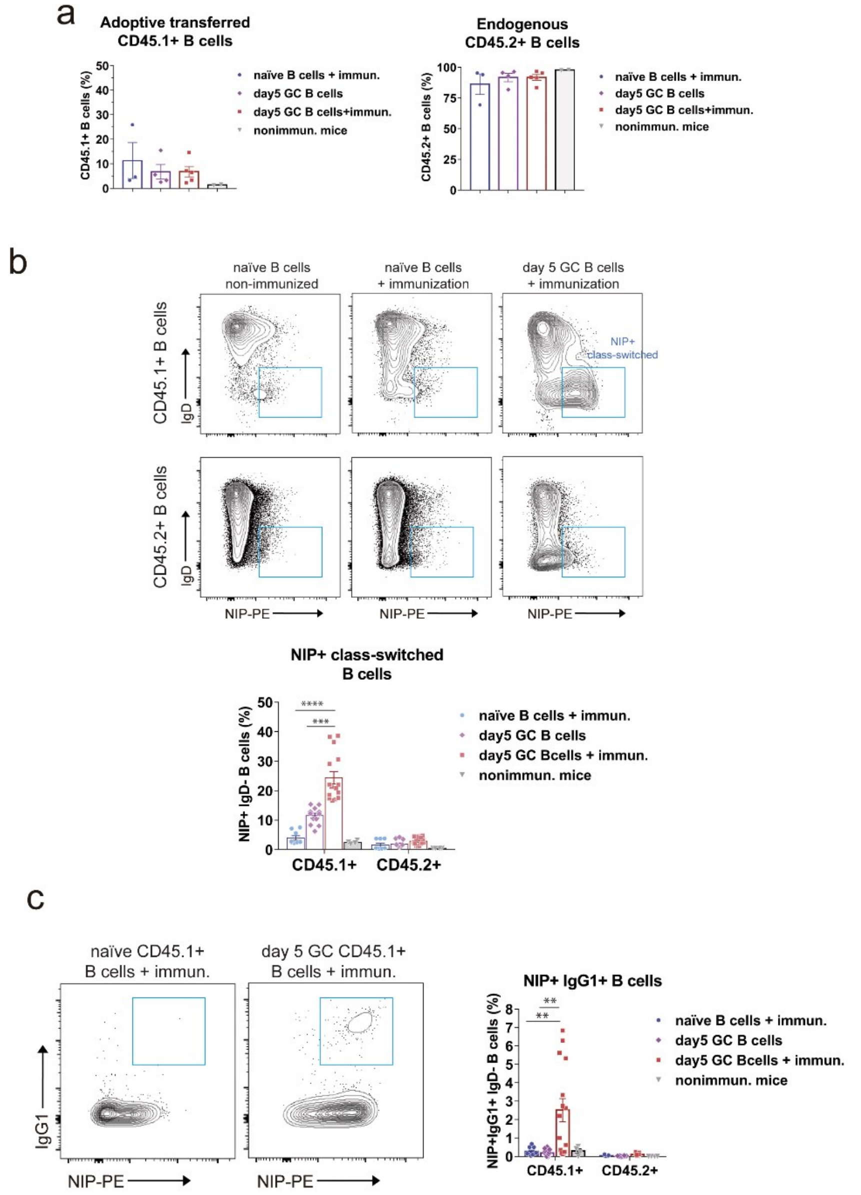

Figure S4. B cells generated in the in vitro GC system progress in vivo to high-affinity antigen-specific B cells in a secondary response. **a,** CD62L+CD44- naïve B cells from B1-8^{low} CD45.1+ knockin mice were purified by cell sorting and inoculated into CD45.2+ wild type C57BL/6 mice. Likewise, B cells from day 5 GC in vitro cultures of B1-8^{low}/OT-2 cells were isolated and inoculated into CD45.2+ wild type C57BL/6 recipient mice. Thirty days after cell inoculation, mice were i.p. immunized with NP-CGG+alum and the spleens were extracted and analyzed seven days later. Bar plots show the percentages of CD45.1+ and CD45.2+ B cells in the spleens of n=3 mice per group. Non-immunized C57BL/6 mice were used as negative controls. **b,** Contour plots of spleen CD19+ B cells from mice adoptively transferred with either naïve B cells or B cells from day 5 in vitro GC cultures and immunized or not with NP-CGG+alum. The plots show the analysis of IgD expression and NIP-FITC binding. Quantitative data corresponding to 4-5 mice per group analyzed in triplicate are shown in the bar plots below. Data represent the mean ± s.e.m. ***, p<0.001; ****, p<0.0001 (two-way ANOVA test). **c,** Contour plots of spleen CD19+ B cells from mice adoptively transferred with either naïve B cells or B cells from day 5 in vitro GC cultures and immunized with NP-CGG+alum. The analysis of IgG1 expression and NIP-PE binding was carried out on CD45.1+CD19+IgD- B cells. Quantitative data for both CD45.1+ and CD45.2+ cells are shown in the bar plots below. Data represent the mean ± s.e.m. **, p<0.01 (two-way ANOVA test).

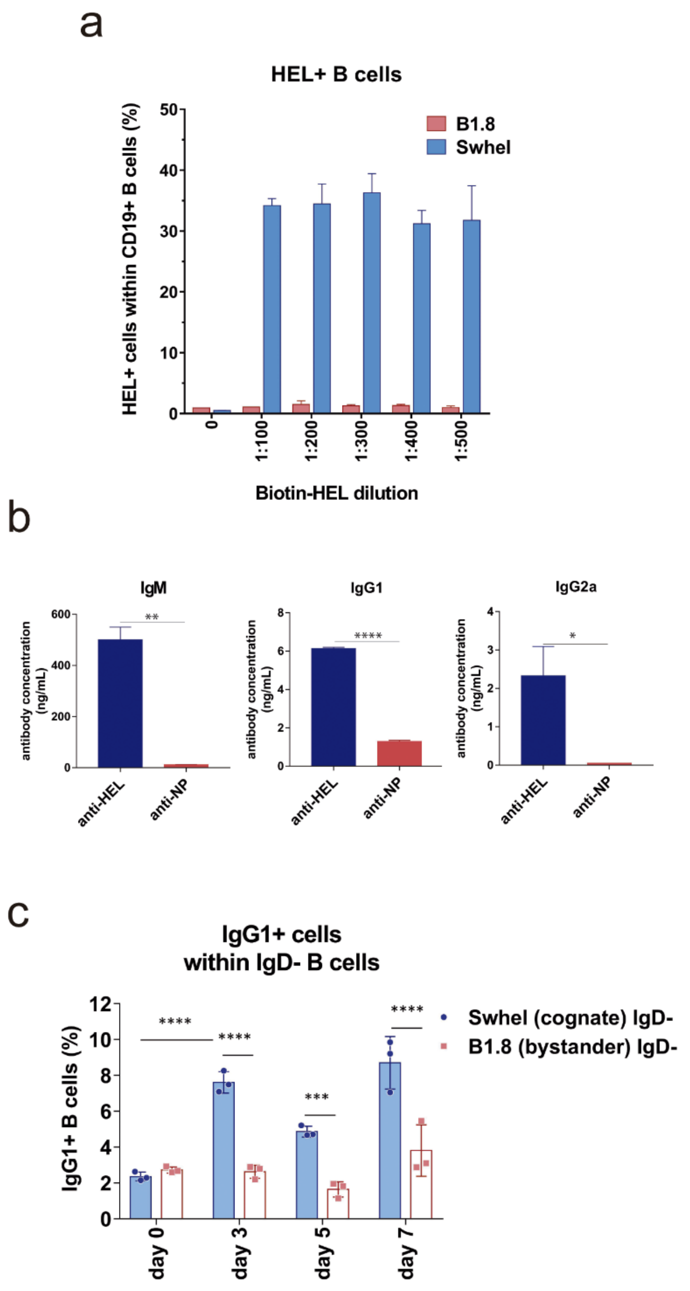

Figure S5. a, Estimation of the percentage of HEL-binding B cells within the CD62L+CD44- naïve CD19+ B cell population in spleens of SWHEL knockin mice. Spleen cells were incubated with the indicated dilutions of HEL-biotin stock. Spleen cells from B1-8^{low} mice were used as negative controls. Data represent the mean ± s.e.m. ($n = 3$). **b,** Measurement of concentrations of IgM, IgG1 and IgG2a specific for NIP in the supernatant of 7 day co-cultures of B1-8^{low} (bystander), SWHEL (cognate) and OT-2 cells stimulated with beads coated with HEL+OVA (1:0.3 cell/bead ratio) and beads coated with NP-BSA (1:0.3 ratio). Antibody concentrations were measured by ELISA. Data represent the mean ± S.D. *, p<0.05; **, p<0.01; ***, p<0.001 (unpaired t-test). **c,** Bar plot showing the percentage of IgG1+ cells within IgD-CD19+ B cell population of co-cultures of B1-8^{low} (bystander), SWHEL (cognate) and OT-2 cells stimulated with beads coated with HEL+OVA (1:0.3 cell/bead ratio) and beads coated with NP-BSA (1:0.3 ratio) and analyzed at the indicated time points. Data represent the mean ± S.D. ***, p<0.001; ****, p<0.0001 (two-way ANOVA test).

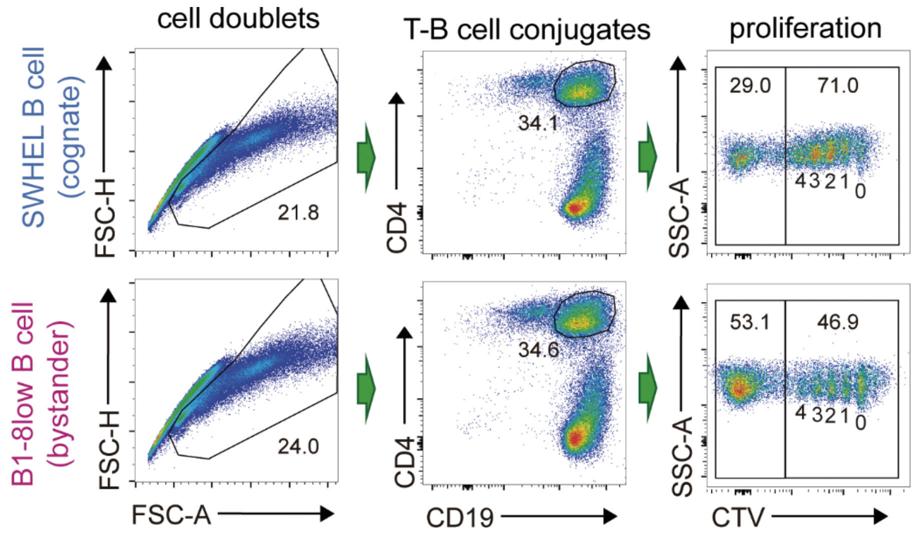

Figure S6. Detection of T-B cell conjugates by flow cytometry. Lymphoid cells were gated according to the Forward Scatter (FSC) and Side Scatter (SSC) signals. Cell doublets were selected within the lymphoid population according to FSC-H and FSC-A as shown. T-B cell conjugates were detected within cell doublets according to the expression of CD19 and CD4 simultaneously. The prior labeling of the either cognate B cells or bystander B cells with CTV helped to identify each cell type within the T-B conjugates.

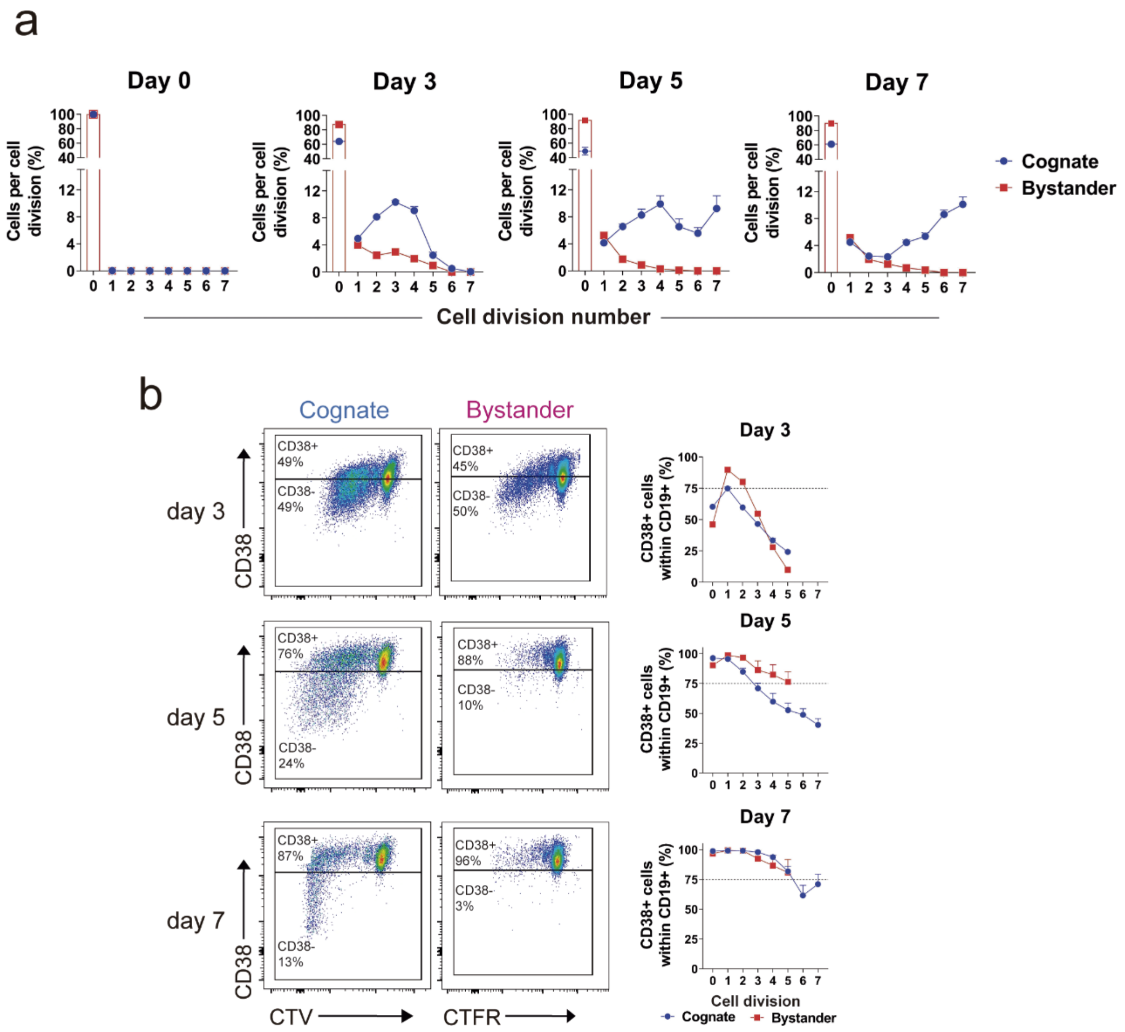

Figure S7. Short proliferative span and early conversion into B cell memory of bystander B cells. **a,** These data represent all the time points of the experiment shown in Fig. 8a. **b,** These data represent all the time points of the experiment shown in Fig. 8c.

Figure 2d- phospho-Akt

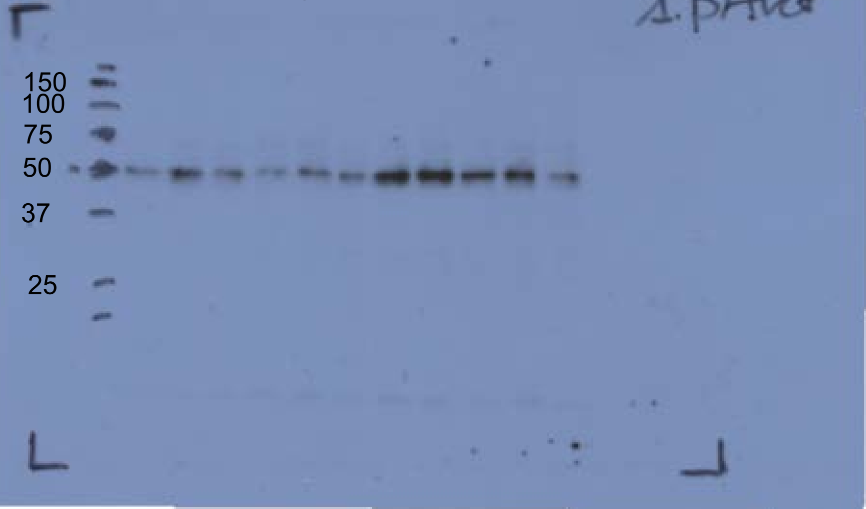

Figure 2d- phospho-ERK

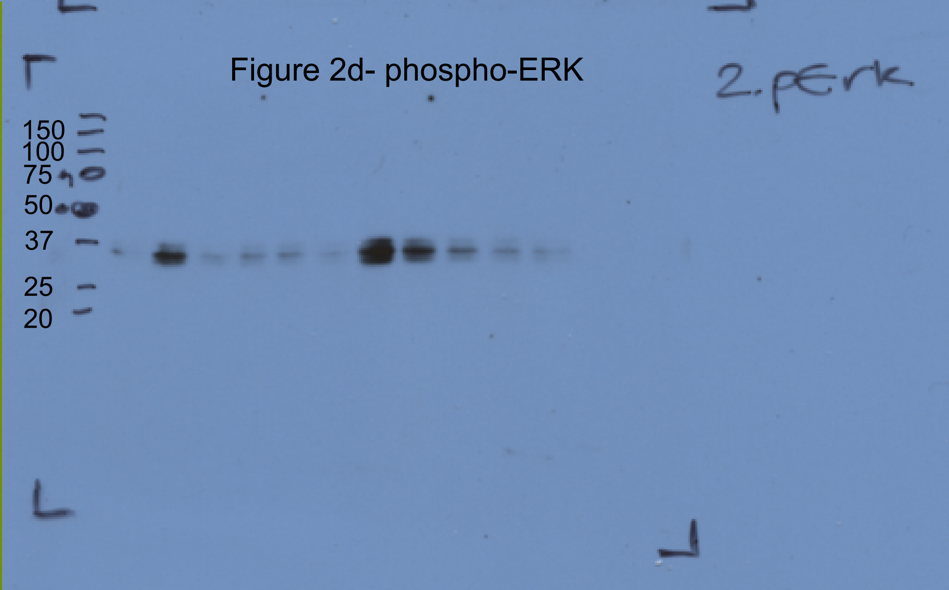

Figure 2d- Actin

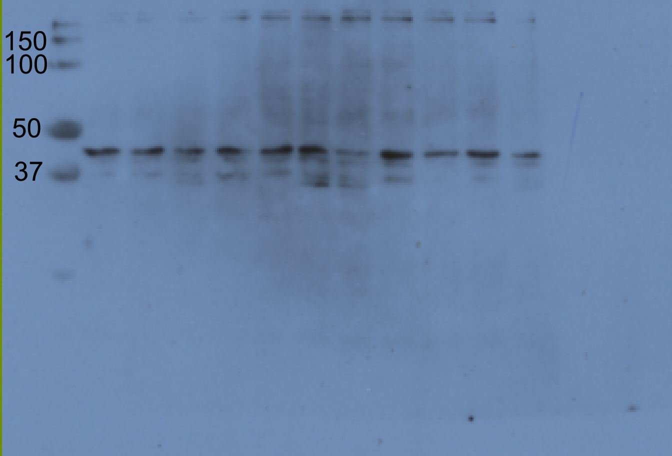

Figure 2d- phospho-Syk

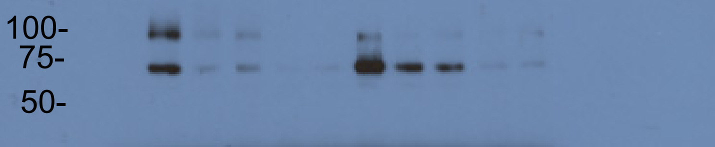

Figure 2d- Actin

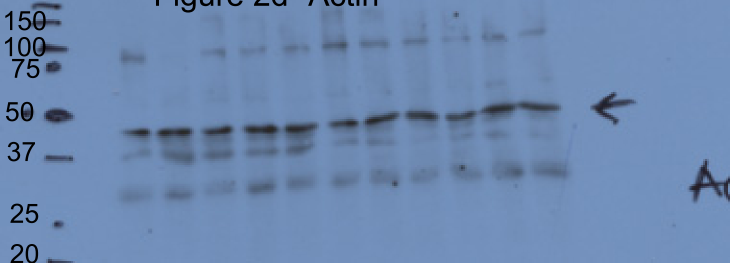

Figure 2d- phospho-Ig-alpha

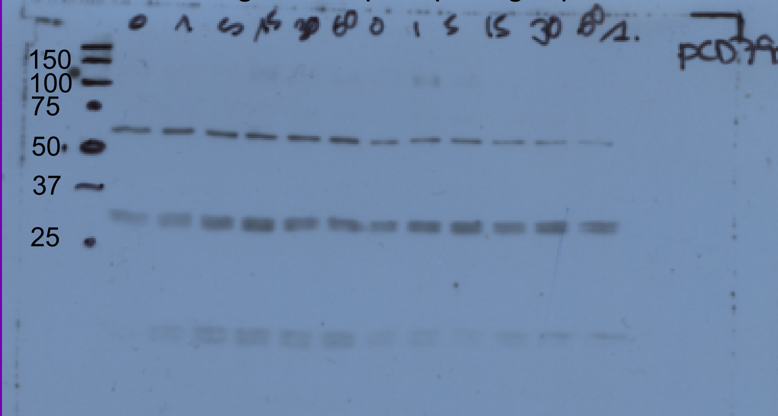

Figure 2e- phospho-Akt

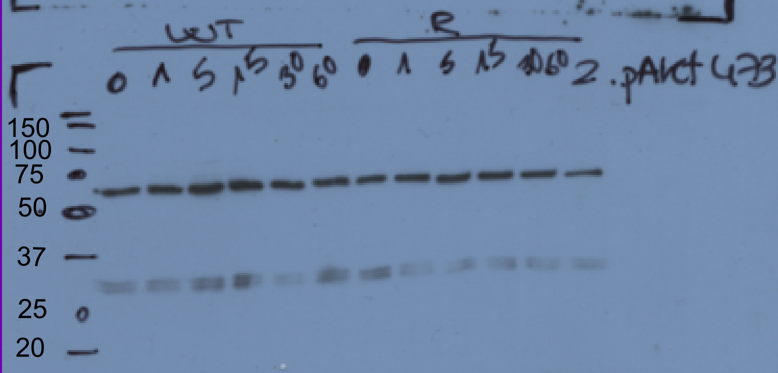

Figure 2e- phospho-ERK

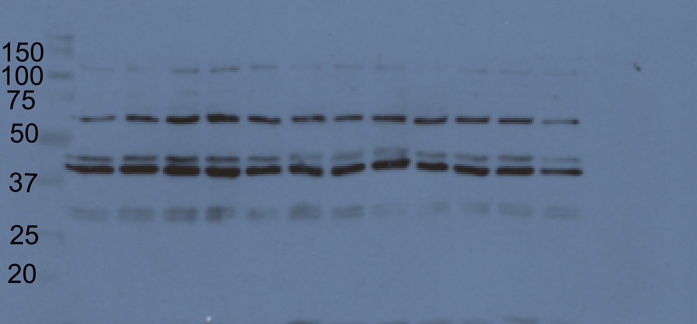

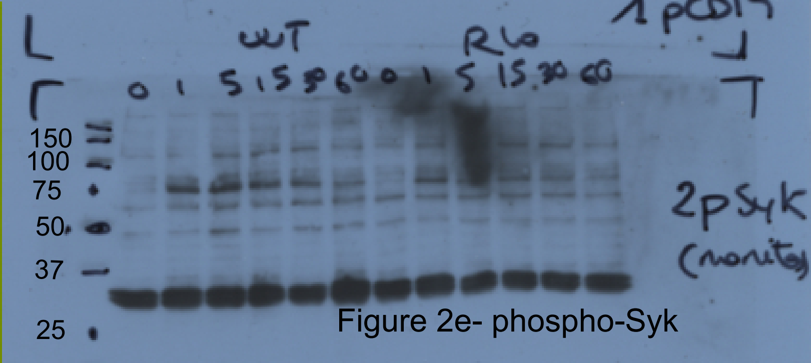

Figure 2e- phospho-Syk

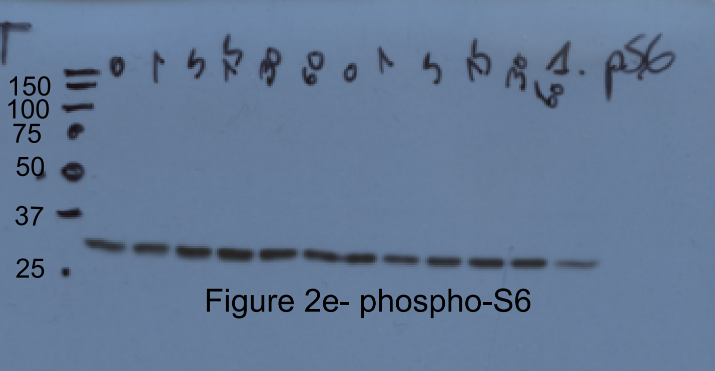

Figure 2e- phospho-S6

Figure 2e- Actin

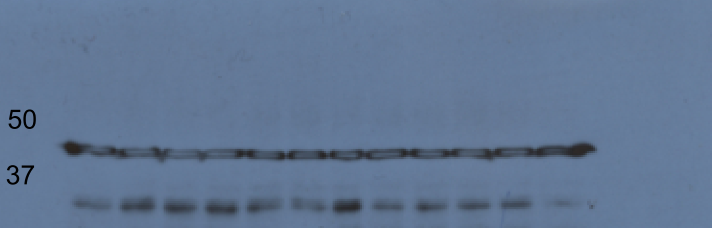

Figure S8. Uncropped blot images for Fig. 2d and 2e. The specificity of the antibody used for western blot is identified in each panel. Precision Plus Portein Dual Color Standards (Bio-Rad) were used as markers. Films were positioned on top of the blotting membrane with the colored markers and positions were hand-labeled.

Supplementary Table 1

REAGENT or RESOURCE	SOURCE	IDENTIFIER	Dilution
Antibodies			
Anti-Actin (clone AC-74)	Sigma	Cat#A5316	1:1000
Anti-AKT (clone 9272)	Cell Signaling	Cat#9272	1:100
Anti-CD45R APC (clone RA3-6B2)	BD Pharmigen	Cat#553092	1:200
Anti-CD45R APC-Cy7 (clone RA3-6B2)	BD Pharmigen	Cat#552094	1:200
Anti-CD45R Biotin (clone RA3-6B2)	BD Pharmigen	Cat#553085	1:200
Anti-CD45R FITC (clone RA3-6B2)	BD Pharmigen	Cat#553088	1:200
Anti-CD45R V450 (clone RA3-6B2)	BD Pharmigen	Cat#560473	1:200
Anti-Bcl6 647 (clone K112-91)	BD Pharmigen	Cat#561525	1:100
Anti-Bcl6 PE (clone K112-91)	BD Pharmigen	Cat#561522	1:100
Anti-Blimp1 PE (clone 5E7)	BD Pharmigen	Cat#564268	1:100
Anti-CD4 647 (clone RM4-5)	BD Pharmigen	Cat#557681	1:200
Anti-CD4 PerCP (clone RM4-5)	BD Pharmigen	Cat#553052	1:100
Anti-CD4 605 (clone RM4-5)	Biolegend	Cat#100547	1:100
Anti-CD8 Biotin (clone 53-6.7)	BD Pharmigen	Cat#553029	1:200
Anti-CD11c Biotin (clone HL3)	BD Pharmigen	Cat#553800	1:200
Anti-CD16/32 purified (clone 2.4G2)	BD Pharmigen	Cat#553141	1:300
Anti-CD19 PE (clone 1D3)	eBiosciences	Cat#12-0193	1:200
Anti-CD19 PE-Cy7 (clone 1D3)	BD Pharmigen	Cat#561739	1:200
Anti-CD25 APC (clone 3C7)	BD Pharmigen	Cat#557192	1:200
Anti-CD38 AF488 (clone 90)	Biolegend	Cat#102714	1:200
Anti-CD38 APC (clone 90)	Biolegend	Cat#102712	1:200
Anti-CD40 PE-Cy5 (clone 3/23)	Biolegend	Cat#124617	1:200
Anti-CD43 Biotin (clone S7)	BD Pharmigen	Cat#553269	1:200
Anti-CD45.1 APC-Cy7 (clone A20)	BD Pharmigen	Cat#560579	1:100
Anti-CD45.1 PE-Cy7 (clone A20)	BD Biosciences	Cat#560578	1:200
Anti-CD45.2 APC (clone 104)	BD Pharmigen	Cat#558702	1:100

Anti-CD45.2 PerCP-Cy5.5 (clone 104)	BD Biosciences	Cat#552950	1:200
Anti-CD73 PE-Cy7 (clone TY/11.8)	Invitrogen	Cat#25-0731-82	1:200
Anti-CD80 PerCP-Cy5.5 (clone 16-10A1)	Biolegend	Cat#104722	1:100
Anti-CD86 PE-Cy5 (clone GL1)	eBiosciences	Cat#15-0862	1:200
Anti-CD95 FITC (clone Jo2)	BD Pharmigen	Cat#554257	1:100
Anti-CD95 PE-Cy7 (clone Jo2)	BD Pharmigen	Cat#557653	1:200
Anti-CD138 APC (clone 281-2)	BD Pharmigen	Cat#558626	1:100
Anti-CD273 (PDL2) APC (clone TY25)	Biolegend	Cat#107210	1:200
Anti-CD279 (PD1) FITC (clone J43)	eBiosciences	Cat#11-9985-85	1:200
Anti-CXCR4 Biotin (clone 2B11)	eBiosciences	Cat#13-9991-80	1:100
Anti-CXCR5 Biotin (clone 2G8)	BD Pharmigen	Cat#551960	1:100
Anti-F4/80 Biotin (clone BM8)	Biolegend	Cat#123105	1:200
Anti-Gr1 Biotin (clone RB6-8C5)	BD Pharmigen	Cat#553125	1:200
Anti-GL7 647 (clone GL7)	BD Pharmigen	Cat#561529	1:300
Anti-GL7 FITC (clone GL7)	BD Pharmigen	Cat#553666	1:300
Anti-IgD Biotin (clone 11-26c)	eBiosciences	Cat#13-5993-81	1:100
Anti-IgD FITC (clone 11.26c)	BD Horizon	Cat#562022	1:200
Anti-IgD 647 (clone 11.26)	eBiosciences	Cat#51-5993-82	1:200
Anti-IgD V450 (clone 11.26c)	BD Horizon	Cat#560869	1:200
Anti-IgG1 Bv421 (clone A85-1)	BD Biosciences	Cat#562580	1:200
Anti-IgG1 PE (clone A85-1)	BD Pharmigen	Cat#550083	1:200
Anti-kappa Biotin (clone RMK-12)	Biolegend	Cat#407204	1:200
Anti-IgM PE (clone II/41)	eBiosciences	Cat#12-5790-81	1:200
Anti-IgM APC (clone II/41)	eBiosciences	Cat#17-5790-82	1:200
Anti-IgM F(ab')2	Jackson Immunoresearc h	Cat#115-006-075	10μl/mL (described in methods)
Anti-NK1.1 Biotin	BD Pharmigen	Cat#553163	1:200
Anti-pAkt (S473) (clone D9E)	Cell Signaling	Cat#4060	1:100 (FACS)<br>1:1000 (WB)

Anti-pCD79α (Y182)	Cell Signaling	Cat#5173	1:1000 (WB)
Anti-pErk (T202/Y204)	Cell Signaling	Cat#9101	1:100 (FACS)<br>1:1000 (WB)
Anti-pS6 (S240/244) (cloneD68F8)	Cell Signaling	Cat#5364)	1:100 (FACS)<br>1:1000 (WB)
Anti-pSYK (Y525/Y526)	Cell Signaling	Cat#2711	1:100 (FACS)<br>1:1000 (WB)
Anti-Vα2 PercP/Cy5.5 (clone B20.1)	Biolegend	Cat#127813	1:200
Chemicals, Peptides, and Recombinant Proteins			
Polybead Carboxylate 1 μm	Polysciences	Cat#08226-15	Methods and Figure Legend
FluoSpheres Carboxylate 1 μm Crimson (625/645)	ThermoFischer	Cat#F8816	Ratio 3 Beads: 1 B cell
Fluoresbrite Carboxylate 1 μm Y/G (441/486)	Polysciences	Cat#15702	Ratio 3 Beads: 1 B cell
Cell Trace Far Red Nuevo	Thermo Fischer	Cat#C34564	1:1000
Cell Trace Violet	Life Technology	Cat#C34557	1:1000
DAPI	Merck	Cat#268298	1:1000
Ghost Dye 540	TONBO	Cat# 13-0879-T100	1:1000
Ghost Dye 780	TONBO	Cat#13-0865-T100	1:1000
NIP(7)-BSA	Biosearch Technology	Cat#N-5050L-10	10μl/mL
NIP(41)-BSA	Biosearch Technology	Cat#N-5050H-10	10μl/mL
NIP(15)BSA-FITC	Biosearch Technology	Cat#N-5040F-10	1:100
NP(36)-PE	Biosearch Technology	Cat#N-5070-1	1:100
NIP-OVAL	Biosearch Technology	Cat#N-5041	Methods and Figure Legends
Phalloïdin-TRICT	Sigma	Cat#P-1951	1:400

Phalloïdin-647	ThermoFisher	Cat#A-22287	1:400
Streptavidin-PercP	BD Pharmigen	Cat#554064	1:500
Streptavidin-APC	BD Pharmigen	Cat#554067	1:300
Streptavidin-APC-Cy7	BD Pharmigen	Cat#554063	1:300
Streptavidin-605	BD Pharmigen	Cat#563260	1:100
IL-4	Peprotech	Cat#214-14	5 ng/ml
Albumin from chicken egg white	Sigma	Cat#A5503	Methods and Figure Legends
Lysozyme from chicken egg white	Sigma	Cat#L4919	Methods and Figure Legends
