# Supplementary material for: Recreation of an antigen-driven germinal center in vitro by providing B cells with phagocytic antigen
Source: Commun Biol. 2023 Apr 20;6:437. doi: 10.1038/s42003-023-04807-0 (PMC10119099; doi:10.1038/s42003-023-04807-0)
Supplement: Supplementary file 3 — DEscription of Additional Supplementary Files [file 42003_2023_4807_MOESM3_ESM.pdf]

Description of Additional Supplementary Files

-**Figure S1.** Generation of a GC reaction by phagocytic B cells and helper T cells does not require a third cell type.

- **Figure S2.** B cells undergo Ig class switching *in vitro* when stimulated with bead-bound antigen regardless the original affinity of the BCR expressed by naïve B cells.

- **Figure S3.** Generation of somatic mutations in IgH V genes in conditions of GC formation *in vitro*.

- **Figure S4.** B cells generated in the in vitro GC system progress in vivo to high-affinity antigen-specific B cells in a secondary response.

- **Figure S5.** Estimation of the percentage of HEL-binding B cells in spleens of SWHEL knockin mice, and measurement of concentrations of IgM, IgG1 and IgG2a specific for NIP in the supernatant of 7 day co-cultures.

- **Figure S6.** Detection of T-B cell conjugates by flow cytometry.

- **Figure S7.** Short proliferative span and early conversion into B cell memory of bystander B cells.

-**Figure S8.** Uncropped blot images for Fig. 2d and 2e**.**

Supplementary Data 1. Gene expression data found in Affimetrix microarrays for GC cells in vivo, GC cells produced in vitro, LPS-stimulated B cells and resting naïve B cells.

Supplementary Data 2. Source data underlying the graphs and charts presented in the main figures.
